# Self-Propulsion and a Push–Pull Mechanism in Sessile Droplets

**DOI:** 10.1021/acs.langmuir.5c01246

**Published:** 2025-06-11

**Authors:** Robab Jahangir, Yewon Kim, Vahid Nasirimarekani

**Affiliations:** † 28283 Max Planck Institute for Dynamics and Self-Organization, Am Fassberg 17, 37077 Göttingen, Germany; ‡ Laboratory of Fluid Physics and Biocomplexity, Am Fassberg 17, 37077 Göttingen, Germany

## Abstract

Self-propelled droplets on solid substrates can autonomously move by physical forces, such as surface tension. This phenomenon mimics natural processes, such as the crawling of living cells or the propulsion of oil droplets on water, and provides valuable insights that apply to both natural phenomena and microfluidic applications. In this study, the self-propulsion of the evaporating droplet by the surface tension gradient on a polymer-coated substrate is investigated. We have studied the internal dynamics and mechanism of droplet motion in sessile and 2D-confined droplets. We report that the asymmetric strength of the Marangoni vortices within the sessile droplet results in a push–pull mechanism that propels the droplet on the substrate. Furthermore, in a self-propelling droplet, the interfacial flow in the flattened droplet propagates at the droplet’s free interface toward the droplet back, causing a continuous polar contraction and propulsion of the droplet. These findings provide essential insight into understanding the role of fluid physics in the self-propulsion of active droplets or living organisms.

## Graphical abstract

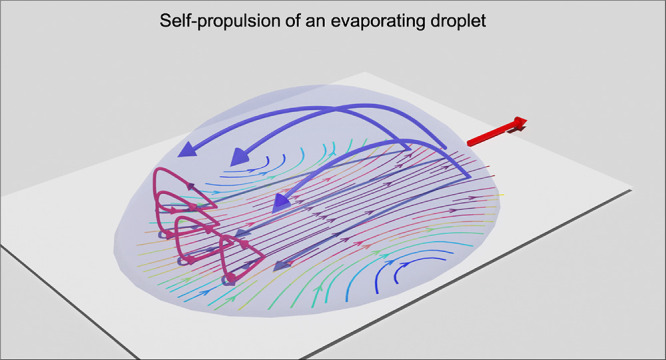

## Introduction

A self-propelled multicomponent droplet, self-driven on a substrate, is an example of an out-of-equilibrium system.^1,2^ Out-of-equilibrium motility is the intrinsic property of life, from single cells to multicellular organisms.^3^ However, the self-propulsion motility in active systems such as living cells has a higher degree of complexity due to continuous energy dissipation.^4^ Therefore, understanding dynamic systems in which only the physical parameters could induce such out-of-equilibrium propulsion is essential. In a nonactive system, the self-propulsion is usually achieved by a gradient of nonzero local forces on the boundary, as exemplified by cells crawling on a surface which push at the front and pull at the back^5,6^ (Figure 1a). Such a nonzero local force can be generated in a nonactive liquid droplet by surface tension gradients, which would lead to the propulsion of the droplet, similar to a living cell.^7,8^

**Figure 1:**
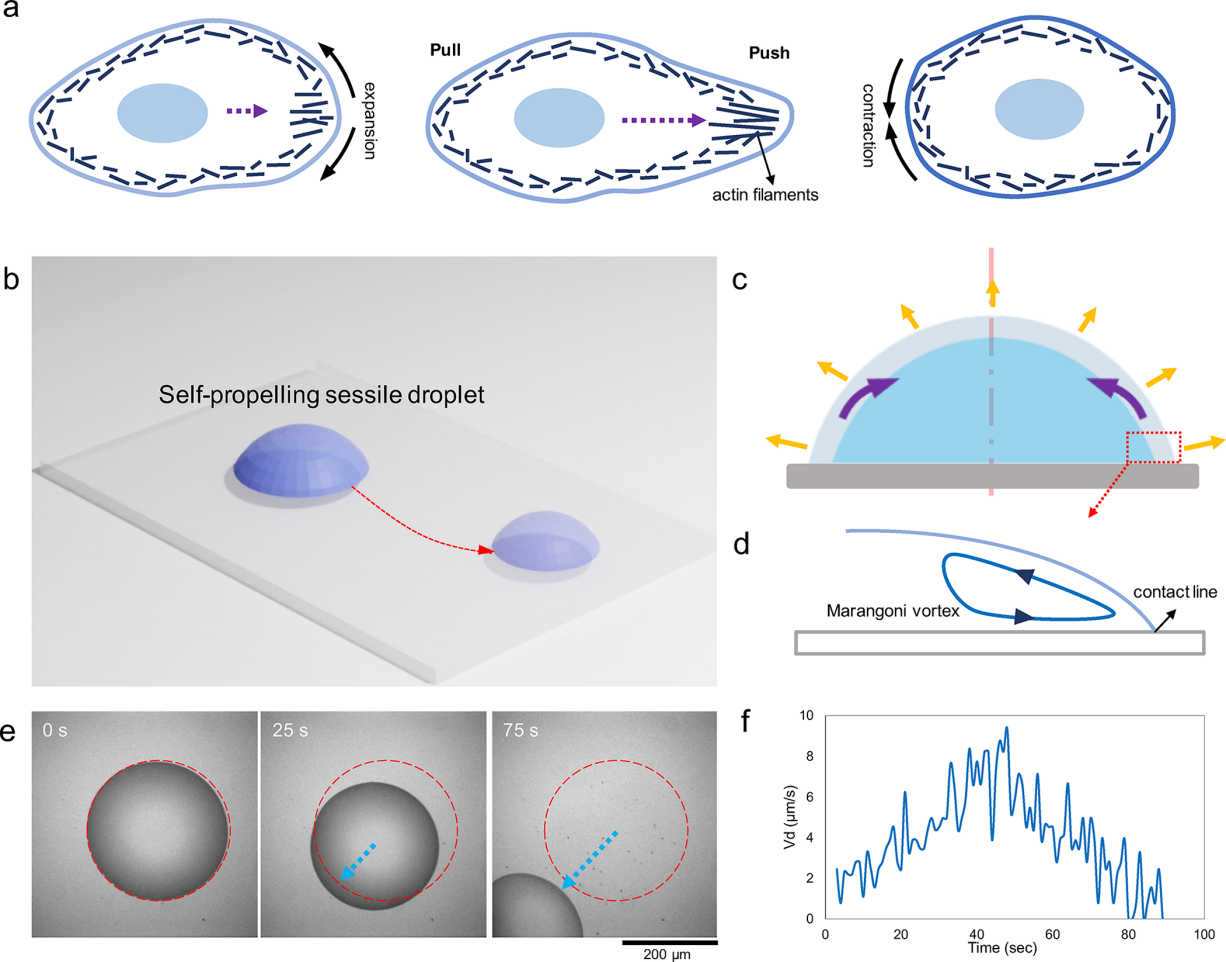
Self-propulsion of a surfactant-laden droplet on a polymer-brushed substrate. (a) Schematic representation of the crawling of living cells as a forward push and backward pull due to the activity of the actin network (inspired by Sadava et al.^32^). (b) Schematics of an evaporating sessile droplet self-propelling on a glass substrate. (c) Mechanism of droplet contraction in a surfactant-laden droplet, which is caused by Marangoni vortices in the vicinity of the contact line. (d) Schematic representation of the Marangoni vortex. (e) Experimental observation of a self-propelling droplet during evaporation (inverted optical microscopy images); red dashed line highlights the initial contact line of the droplet. (f) Velocity magnitudes of a self-propelling droplet showing a nonlinear velocity trend throughout the course of drying.

The self-propulsion of a droplet rooted in surface tension is a fundamental aspect of many natural phenomena, from the movement of oil droplets on water to the flight of insects.^9–12^ In nature, self-propelled droplets are found in various forms, such as locomotion of certain microorganisms, spore dispersal of fungi, and movement of plant cuticles. Besides the fundamental importance of the topic, the self-propelled droplet phenomenon has wide-ranging implications for technological applications, including materials science, biomedicine, and energy production. For instance, studying self-propelled droplets can inspire the design of novel microrobots, bioinspired propulsion systems, and advanced microfluidic devices, which is important in fields such as environmental monitoring, healthcare, and energy harvesting.^7,13^

Self-propelled droplet migration has been investigated on versatile substrates including fluid interfaces such as Leindenfrost-like systems.^12,14^ Liquid marble or droplet dissolution processes have been widely employed in such cases.^12,14–16^ Additionally, motility due to surfactant adsorption on liquid interfaces caused by pH and alkaline-ion gradient has also been reported.^12,17–19^ However, the droplet propulsion on a solid substrate demands a contact line depinning to overcome defects associated with the substrate surface.^20^ Hence, external forces such as concentration gradient, temperature, and electric field are generally required to trigger the droplet motion.^20–23^

In autonomous self-propulsion, without applying external forces, droplet studies are generally investigated on surface-modified glass substrates (Figure 1b). Self-propulsion is imposed on solid substrates by inducing a surface tension gradient on the rear and front of the droplet or by preprinting the wettability gradients on the surface.^9,24^ A number of physio-chemical mechanisms such as the desorption of surfactants at the interface can generate a surface tension gradient,^25^ which eventually results in contact angle hysteresis. Therefore, such a droplet moves toward the side of the droplet with lower surface tension. However, in all of these substrate-based methods, the gradient applied by the surface defines the self-propulsion of the droplet.

Evaporation is a spontaneous phenomenon that occurs naturally in all volatile liquids deposited on a substrate. An evaporating sessile droplet shows a capillary flow of the liquid toward the contact line where the evaporation is faster. This results in a distinctive coffee ring effect.^26,27^ However, when surface tension-reducing agents are added inside the droplet (surfactant-laden droplet), the surface tension gradient becomes negative toward the contact line; therefore, a circulating flow inside the droplet forms, known as Marangoni circulations.^28,29^ The Marangoni vortex can contract the droplet by evaporation toward its center (Figure 1c,d).^30,31^

In this work, we studied a surfactant-laden droplet on a polymer-brushed substrate to investigate how the droplet might self-propel due to continuous evaporation. In other words, can continuous evaporation and contraction of the droplet lead to an autonomous self-propulsion? To facilitate the propulsion of the droplet, the polymer-brushed substrate was used to avoid droplet pinning and allow for smooth contraction of the droplet. By incorporating fluorescent beads into the droplets, we have analyzed the flow dynamics within the droplet, both for a sessile droplet and for a quasi-2D flattened droplet. Here, we report that evaporation of a surfactant-laden droplet can lead to self-propulsion, which is analogous to the push–pull crawling dynamics of living cells in nature. Therefore, our results provide important insights into how the surface tension gradient results in the flow dynamics and propulsion of an evaporating droplet.

## Materials and Methods

### Materials

PEG 6k and 20k and PEO 100k were purchased from Merck, Germany. Fluorescent beads (2 μ*m* in diameter, red fluorescent 580/605) were purchased from Invitrogen. Poly­(l-lysine)-*graft*-poly­(ethylene glycol) (PLL-*g*-PEG) (SuSoS AG, Switzerland) at a final concentration of 0.1 mg/mL in 10 mM 4-(2-hydroxyethyl)-1-piperazineethanesulfonic acid (HEPES) (pH 7.4, at room temperature) was used for surface functionalization. High-resolution glass slides 24 × 55 mm were purchased from Paul Marienfeld GmbH & Co. KG, Germany.

### Sample Preparation

3% PEG (w/w) was dissolved in 1% Milli-Q water using a magnetic stirrer for 3 h. After thorough dispersion of the PEG, a 1/10 volume ratio of fluorescence beads was added as tracer particles in the PEG solution. An additional vortexing for 1 min was done for all samples before conducting the experiments.

### PLL-*g*-PEG Surface Functionalization

The functionalization procedure in order to apply the PLL-*g*-PEG coating on the glass substrates can be divided into the following two steps:

1. *Cleaning:* Glass coverslips were first immersed in 100% ethanol for 10 min and then rinsed with deionized water. Next, they were sonicated in an acetone bath for 15 min, followed by incubation in 96% ethanol for 10 min. Afterward, an additional 2 h incubation was done in a 2% Hellmanex III solution. Finally, an intensive washing was done in deionized water to remove the Hellmanex solution along with drying with filtered pressurized air.
2. *Surface Functionalization:* The cleaned glass slides were first activated by air plasma treated for 30 s at 0.5 mbar. Subsequently, they were incubated in a solution of 0.1 mg/mL poly­(l-lysine)-*graft*-poly­(ethylene glycol) (PLL-*g*-PEG) (SuSoS AG, Switzerland) in 10 mM HEPES, pH 7.4, for 1 h on parafilm (Pechiney, U.S.A.), inside the ventilated hood. Lastly, the coverslips were lifted carefully, and the residual PLL-*g*-PEG solution was removed by air drying. All procedures were performed at room temperature.

### Experimental Setup

#### Sessile Droplet

The evaporation of the sessile droplet proceeds by pipetting 0.3 μL of a solution containing fluorescence particles of size 2 μm on the PLL-*g*-PEG functionalized glass slides. The glass slide with the droplet was then positioned on the microscope stage for imaging. To avoid the interaction of airflow with the evaporating droplet, a translucent container of size 5 × 5 × 2 cm was used to cover the setup.

#### Confined Droplet

For the confined geometry studies, first, a confined space of size 20 × 20 mm was created on the Pll-*g*-PEG-coated glass slide. For this, a double adhesive spacer with 30 and 40 μm heights was strategically placed on top of the glass slide. Second, a 0.3 μL of solution containing fluorescence particles of size 2 μm was pipetted in the center of the confined area, followed by the careful positioning of another glass slide on top of the confined area such that the droplet was confined between two PLL-*g*-PEG fictionalized glass surfaces. The confined system was then positioned on the microscope stage for recording.

### Microscopy

An inverted microscope IX-71 was used for acquiring images. Different objective lenses including 4×, 10×, and 20× (Olympus, Japan) were used according to the experimental configurations. A 200 lm metal arc lamp (Prior Scientific Instruments, U.S.A.) was used for the excitation of the samples. The frames were captured by a high-resolution CCD camera (CoolSnap HQ2, Photometrics) at 1 frame/300 ms and 20 frames/min for sessile and confined droplets, respectively.

### Particle Tracking

To investigate the flow fields, velocity, and displacement of fluorescent particles (2 μm) inside the droplets, particle tracking was done using Trackmate and Manual tracking plugins in Image j.

1. *PTV measurements* Particle tracking velocimetry (PTV) was used to track the flow inside droplets. For this, the Trackmate plugin in image j was employed to identify and track individual particles across successive video frames. In Trackmate, the LoG detector (with estimated object diameters corresponding to the frames/pixels of video) and Simple Lap tracker were utilized. According to the pixels/frame, (depending on the objective lens used for video recording) of the processed videos, linking max distance, Gap-close max distance and Gap-closing max frame gap were adjusted between 2 and 16, 4–17, and 4–17 pixels, respectively. Detected spots were then filtered by using a track displacement filter. The tracked data were then exported for further analysis and plotting in Python.
  For vector field plotting, postprocessing involved removing outliers based on the angular deviation of neighboring vectors. For this, PTV data were interpolated onto a uniform grid with 8-pixel spacing using bilinear interpolation, avoiding a 1-pixel resolution grid to optimize computational cost. Subsequently, the uniform grid velocity field was averaged using 16 × 16-pixel kernels for plotting results in Python.
2. *Manual tracking* Manual tracking was done to measure the droplet velocity and to track individual particles wherever required. For this, an individual particle was tracked step-by-step manually in progressive video frames. For each data set, the average velocity was determined by tracking and analyzing four individual particles.

### Contact Angle and Surface Tension Measurement

The contact angle measurements were conducted by side view image recordings. A digital camera (27 fps; Point Gray Grasshopper2) equipped with a telecentric lens (1.0×; working distance: 62.2 mm; Thorlabs Bi-Telecentric lens) and a collimated light source were used to record the side-view images.

The surface tension measurements were conducted by the pendant drop method.^33^ For each solution, the surface tension of 8 drops of 2.5 μL was measured (in room conditions, *T* = 20 °C, RH = 45%). Ten images were collected for each drop in 1 s of recording time. The surface tensions were calculated as an average of these measurements with an average error of 0.14 mN/m.

## Results and Discussion

Evaporation of the droplet containing polyethylene glycol (PEG) on the glass substrate coated with poly-L-lysine backbone and polyethylene glycol (PLL-*g*-PEG) brushes was studied at room temperature (see Methods). The results show that some sessile droplets are self-propelled on the polymer brushes in random orientation. At the same time, some remain pinned and contract over time toward the center of the mass (Figures 1e and S1). In addition, the propulsion velocity shows a nonlinear trend throughout evaporation (Figure 1f).

The droplets are exposed to the surrounding air, which leads to nonhomogeneous or asymmetric evaporation around the droplet. When the evaporation rate is uneven across the droplet, an imbalance in the surface tension forces arises. As a result, the side of the droplet that evaporates faster has a higher surface tension than the side that evaporates more slowly. The asymmetric surface tension therefore results in net forces acting on the droplet,^34^ causing it to move toward the side with slower evaporation.

Although the onset of self-propulsion of the droplet is understandable due to the asymmetric surface tension forces, the internal fluid dynamics and overall shape of such a droplet are not known. In this regard, we have incorporated fluorescent beads into the droplets to understand the fluid dynamics inside the self-propelling droplets. This ought to answer the question of how an unpinned, sessile droplet shrinks in volume and simultaneously self-propels.

### Asymmetric Marangoni Vortices Propel the Droplet

The flow dynamics inside the droplet show the existence of asymmetry of Marangoni vortices in a self-propelled droplet (Figure 2a, Video S1). The asymmetry in the strength of the Marangoni vortices along the droplet leads to either a polar contraction of the droplet or self-propulsion on the substrate (Figure S1). In a self-propelled droplet, the Marangoni vortices (stronger at one side of the droplet) function as a propeller of the liquid volume inside the droplet, which results in propelling the droplet forward (Figure 2b). The propelling function of the Marangoni vortices is analogous to a mechanical propeller (a fan) that recirculates the liquid volume at the vicinity of the droplet interface.^35^

**Figure 2:**
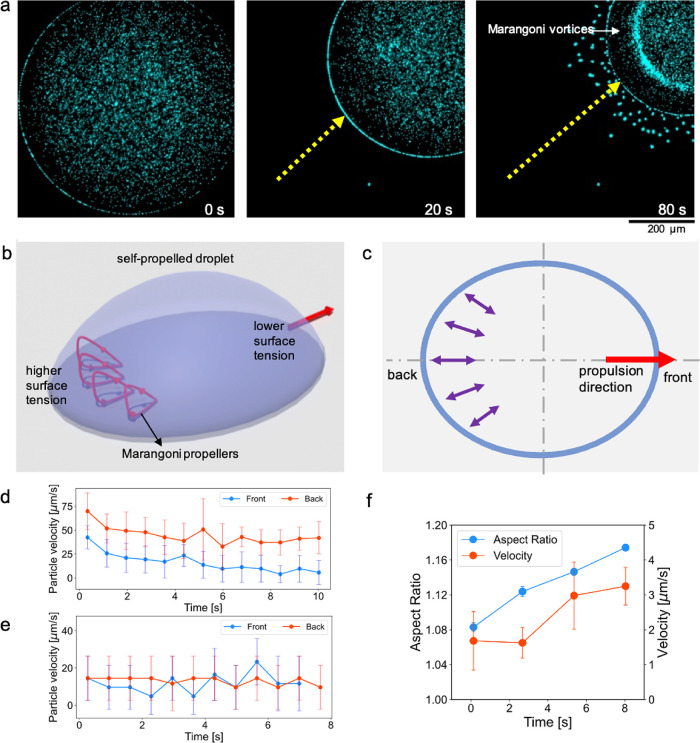
Presence of axisymmetric vortices inside the self-propelled sessile droplet. (a) Time-lapse images of a self-propelled droplet showing a clear band region where the Marangoni vortices occur. (b, c) 3D and 2D schematics of the droplet with polar Marangoni vortices, which act as a propeller to move the droplet forward toward the end with lower surface tension. (d, e) Velocity magnitudes of the droplet contact line at the back and front lines of the droplet for two droplets with average propulsion velocities of 45 and 15 μm/s, respectively. (f) Graph of measurements of the aspect ratio in a self-propelled droplet in relation to the propulsion velocity.

Prior to self-propulsion, the surface tension gradient along the droplet, which is caused by nonuniform evaporation, leads to the formation of the asymmetric Marangoni vortices along the droplet. PEG, which acts as a surfactant, tends to lower the surface tension inside an evaporating droplet (Table S1). Nonuniform evaporation results in relatively higher surface tension on one side of the droplet. PEG then flows toward that side to lower the surface tension, resulting in stronger Marangoni vortices on the same side (Figure 2b). These vortices serve as a propeller; when the force exerted by the propellers is sufficient, the droplet is pushed forward and set in motion. The persistent propulsion of the droplet due to continuous evaporation maintains the droplet in a nonequilibrium state and sustains the self-propulsion until the end of the drying process. Nevertheless, the self-propulsion can be halted due to disturbances caused by the local pinning of the droplet front.

We measured the velocity of the droplet interface at the back and front as the droplet self-propels (Figure 2c). Here, the side of the droplet where the Marangoni propellers are located is considered as the droplet’s back and the other side is considered as the droplet’s front. The back side is specified by the formation of an incomplete coffee ring,^26^ which forms due to particle accumulation as the result of a higher evaporation rate. The velocity magnitudes at the back are relatively higher compared with the droplet front (Figure 2d). However, when the droplet moves slower, the velocity magnitudes appear to be in a similar range (Figure 2e). We assume that the displacement at the back of the droplet is higher as the back of the droplet contracts more due to higher evaporation compared with the front part, which highlights the necessity of studying the flow dynamics inside the droplet. Along the same line, a comparison of the self-propelled droplets with the pinned droplets indicates that the self-propelled droplets generally have an ellipsoidal shape. The pinned droplets have either a circular or an irregular shape but not an ellipsoidal shape. The aspect ratio of the ellipse for self-propelled droplets shows that the ellipse aspect ratio increases as the droplet self-propels faster (Figure 2f). This is plausible as acceleration elongates the droplet along the propulsion direction, which is the long axis of the ellipse. In contrast, the slower the propulsion, the closer the shape approximates a circular shape. In summary, the elongation of the droplets in the form of an ellipse points to the role of internal flows along the longitudinal axis of the droplet, which will be discussed in the next section.

### Internal Flow inside the Self-Propelled Sessile Droplet Reveals a Push–Pull Mechanism

The streamlines inside the self-propelled droplet show that the liquid moves from the back of the droplet to the front inside a self-propelling droplet (Figure 3a,b). Moreover, the velocity magnitudes are higher in the middle part of the droplet compared to the droplet sides, which suggests the path of least resistance for the liquid to be pushed forward (Figure 3b, Video S2). Accordingly, the overall distance that particles travel in the center of the droplet is higher compared to the droplet sides (Figure 3c). Therefore, two different dynamics are observed inside the droplet: (i) a continuous flow in the center, flowing from the droplet back to the droplet front, and (ii) a push–pull flow on the sides of the droplet (Figure 3d). The combination of these two dynamics drives the liquid volume forward toward the droplet front.

**Figure 3:**
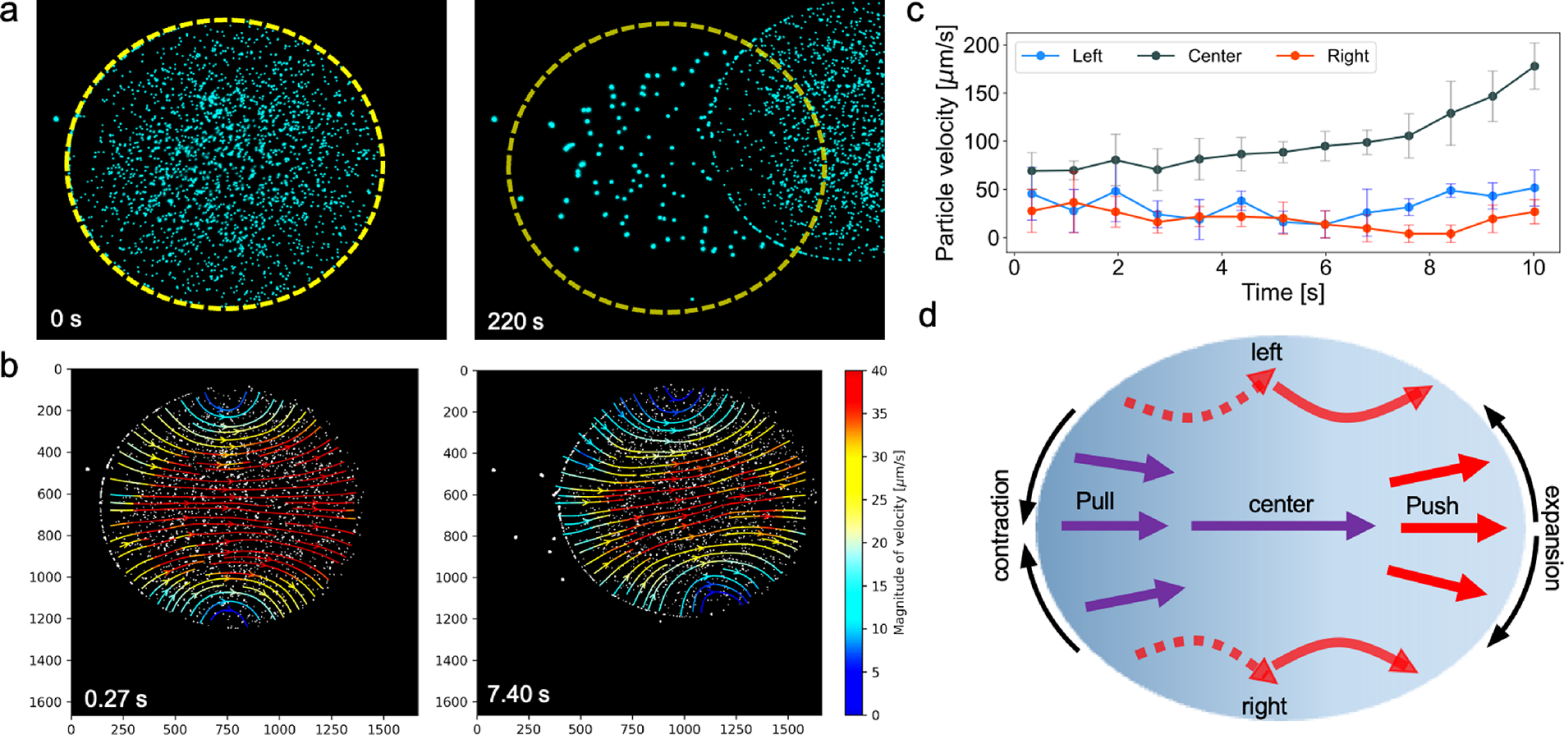
Internal flow dynamics inside the self-propelled sessile droplet triggered by nonuniform evaporation and driven by axisymmetric Marangoni flows. (a) Microscopy images of a self-propelling droplet; the yellow dashed line represents the initial contact line of the droplet. (b) Streamlines colored by velocity magnitude inside the self-propelling droplet at different time frames. (c) Displacement measurements of the sample particles in the center of the droplet and droplet sides which compare the overall distance that particles travel throughout the droplet self-propulsion. (d) Schematics demonstrating the flow dynamics inside the droplet, highlighting the push–pull mechanism, which expands the droplet at the front and contracts it at the back.

The self-propulsion of the evaporating sessile droplet indicates the presence of a contraction–expansion mechanism. As shown previously, higher displacement of the droplet’s back is due to contraction of the droplet at the back. This is the result of higher evaporation and stronger Marangoni flows which contract the interface at the droplet’s back (Figure 2d). Therefore, in the course of evaporation, the entire droplet shrinks over time in a nonlinear trend (measured for the nonpropelled droplets, Figure S2). On the other end, the droplet front is pushed from the rest of the droplet as the liquid volume flows to the droplet front (Figure 2c). This results in an overall push–pull mechanism, in which the pull is due to contraction at the back to reduce the surface area and push is the result of expansion of the surface area at the droplet front.

The liquid on the sides of the droplet does not show a continuity of the motion; it is first pushed out toward the interface and then pulled by the droplet back as the droplet travels along the substrate. This leads to the observation of a push–pull dynamic (Figure 3d, the solid line represents the push dynamic and the dashed line the pull dynamic). This shows that the continuous flow in the middle section of the droplet does not directly affect the liquid at the droplet sides; rather, the liquid at the droplet sides moves due to the overall propulsion of the droplet. Thus, the droplet volume is transported on the substrate by the coupling of these two flows: the continuous and the push–pull flows.

Regarding the overall shape of the self-propelling droplet, we discuss that the droplet exhibits contact angle hysteresis due to the asymmetric Marangoni vortices. Although it is challenging to measure the contact angle of the self-propelling droplet and quantify the contact angle hysteresis, it is well understood that if the contact angle hysteresis exceeds a critical threshold, the droplet will move and then be pushed forward.^36,37^ In our previous work, we observed that the Marangoni vortices increase the apparent contact angle;^38^ the angle increases by about 30% when the strength of the Marangoni vortices reaches its maximum level. Hence, we conclude that an asymmetry in Marangoni vortices means a higher contact angle at the droplet back compared to the front.^39^

Although the fluid flow reveals the self-propulsion dynamics of the droplet, the presence of Marangoni vortices at the droplet back indicates that PEG should continuously flow toward the droplet back in a circulatory flow pattern.^40–42^ However, seemingly, the flow of PEG molecules toward the droplet back does not affect the internal flow inside the droplet. In a pinned sessile droplet, PEG would flow backward through the top interface of the droplet (Figure 4a).^35^ Thereby, we hypothesized that confinement of the droplet between two surfaces and the formation of a quasi-two-dimensional droplet, which can lead to slower propulsion of the droplet (Figure 4b), can be used to observe the interfacial flow. In such an arrangement, the flow of PEG to the droplet back can be visualized.

**Figure 4:**
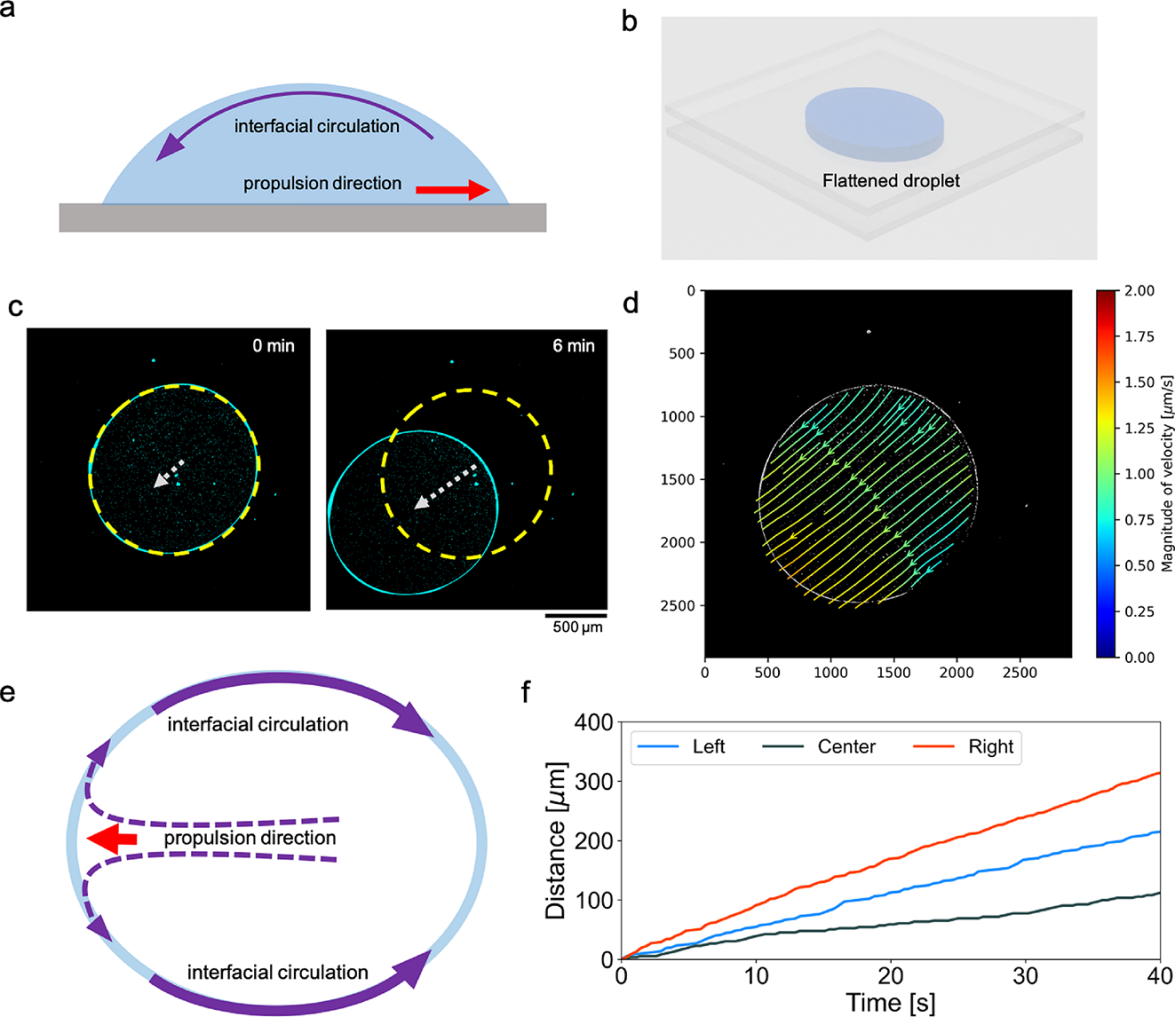
Interfacial flow is observed in a quasi-2D droplet. (a) Schematics demonstration of the interfacial circulation in a self-propelling sessile droplet. (b) 3D schematics of the flattened droplet in between two glass slides. (c) Microscopy images of an example flattened droplet self-propelling over time. (d) Streamlines colored by velocity magnitude inside the example self-propelling droplet at a given time frame. (e) Schematic demonstration of the interfacial flow to the back side of the droplet where the surface tension is higher. (f) Plot showing a comparison of the displacement of tracked single particles in the middle of the droplet and particles on the left and right side of the droplet.

### Interfacial Circulation in a Self-Propelling Droplet

We have flattened droplets between two glass substrates, functionalized with polymer brushes facing each other (Hele-Shaw cell,^43^ Figure 4b), to observe the interfacial flow at the free boundaries of the droplet (the droplet interface at the periphery, in between two flattening surfaces). In addition, the flattened droplet offers the opportunity to investigate whether Marangoni vortices form inside the flattened droplets.

The flow dynamics inside the self-propelled flattened droplet indicate an interfacial flow (at the free boundary of the droplet) from the droplet front toward the droplet back (Figure 4c,d). The streamlines within the flattened droplet reveal a relatively straight and homogeneous flow along the propulsion direction of the droplet (Figure 4d, Videos S3 and S4). Upon reaching the droplet front, some of the particles cling to the interface and move toward the back of the droplet. Manual particle tracking data revealed that the particles at the droplet interface moved at a higher speed and showed a larger displacement than particles in the center of the flattened droplet (Figure 4f), which led to an accumulation of particles at the droplet back.

The backward interfacial flow is observed due to the higher surface tension at the droplet’s back. The self-propulsion in the flattened droplet is triggered by the difference in height between the back and the front of the droplet. The height difference causes contact angle hysteresis, and the droplet moves spontaneously toward the droplet front similar to the self-propulsion in the sessile droplet. The PEG molecules move at the free interface of the droplet toward the droplet back to lower the surface tension. Due to the lubricant properties of the polymer brushes on the glass substrates,^44^ the droplet is unpinned and can freely move between the two glasses. Moreover, due to the evaporation, further surface tension imbalance between the front and back of the droplet appears. Accordingly, PEG continuously flows at the interface toward the droplet’s back to balance the surface tension. Unlike the sessile droplet, due to the 2D constraint, no visible Marangoni vortices were observed. The flattening restricts the spatial freedom so that vortices do not form at the back of the droplet.

In a pinned flattened droplet, in which the droplet front is pinned at a point and the droplet forms a teardrop shape (Figure S3, Video S5), the combination of the central flow and the interfacial flow forms two vortices at the fixation point. The particles accumulate at the back of the droplet, and the droplet continuously contracts from the back. This indicates that the backward flow at the interface mainly serves to reduce the surface area at the back, which contributes to contraction of the droplet. The contraction then leads to a forward flow in the center of the droplet. Nevertheless, there is no forward movement of the pinned droplet. Therefore, the experiments with flattened droplets highlight the importance of interfacial flow, which propagates from the front to the back of the droplet.

## Conclusion

Here, we discuss that the asymmetric Marangoni vortex in an evaporating sessile droplet can function as a propeller on one side of the droplet. The fluid flow generated by the Marangoni propeller can drive an unpinned sessile droplet. By studying the fluid dynamics inside the droplet, we showed that the propulsion is the result of a push at the front and a pull at the back of the droplet. The pulling is the result of a continuous contraction at the back of the droplet to reduce the surface tension by decreasing the surface area. When the fluid flow moves toward the front of the droplet, a pushing mechanism is induced. This resembles the crawling of living cells in both the pushing and pulling mechanisms and the contact angle hysteresis. Furthermore, we have shown that the same droplet, when flattened, exhibits a flow at the interface from the droplet’s front to the back of the droplet. In both cases, continuous evaporation and asymmetric surface tension lead to nonequilibrium dynamics and consequently to continuous self-propulsion. In addition to the fundamental insights gained from the experimental work, we foresee that the understanding of the dynamics of self-propulsion will provide important insights for the development of microfluidic systems in which noninvasive self-propulsion of the droplet is desired. Additionally, this study suggests that fluid physics may play a role in the propulsion of living organisms on a substrate.

## Supplementary Material













## Notes

Asymmetry in the strength of the Marangoni vortex in a self-propelled droplet (ZIP)
Streamlines of the flow inside a self-propelled, sessile droplet (ZIP)
Time-lapse images of the 2D-flattened droplet self-propelling between two surfaces (ZIP)
Streamlines of the flow inside a self-propelled, 2D flattened droplet (ZIP)
Time-lapse images of a pinned 2D-flattened droplet contracting between two surfaces (ZIP)
Comparison of sessile droplets undergoing evaporation on a polymer-brushed glass substrate, comparison of a pinned droplet with a self-propelled droplet; contact angle and surface tension measurements of water and PEG droplets; contraction ratio of the droplets for different PEG molecular weights such as PEO 100k and PEG 20k and 6k; and internal flow in a flattened, pinned 2D droplet that contracts between two polymer-brushed glass substrates during the drying process (PDF)
