# Supplementary material for: Self-Propulsion and a Push–Pull Mechanism in Sessile Droplets
Source: Langmuir. 2025 Jun 11;41(30):19698–705. doi: 10.1021/acs.langmuir.5c01246 (PMC12333417; doi:10.1021/acs.langmuir.5c01246)
Supplement: Supplementary file 6 [file la5c01246_si_006.pdf]

Supporting Information

Self-propulsion and a push-pull mechanism in sessile droplets

Robab Jahangir[1,2], Yewon Kim[1,2], and Vahid Nasirimarekani[1,2*]

[1] Max Planck Institute for Dynamics and Self-Organization, Am Fassberg 17, 37077, Göttingen, Germany

[2] Laboratory of Fluid Physics and Biocomplexity, Am Fassberg 17, 37077, Göttingen, Germany

Corresponding author: vahid.nasirimarekani@ds.mpg.de

Number of pages: 6
Number of figures: 3
Number of tables: 1
Number of videos: 5

Table of Contents

1. Comparison of the pinned and self-propelling sessile droplets (Figure S1)……….. 2
2. Contact angle and surface tension measurements (Table S1)……………………… 3
3. Contraction ratio of the droplet in relation to PEG chain length (Figure S2)………. 4
4. Internal flow in a 2D flattened pinned droplet (Figure S3)…………………………… 5
5. List of the supporting videos……………………………………………………………….. 6

Comparison of the pinned and self-propelling sessile droplets

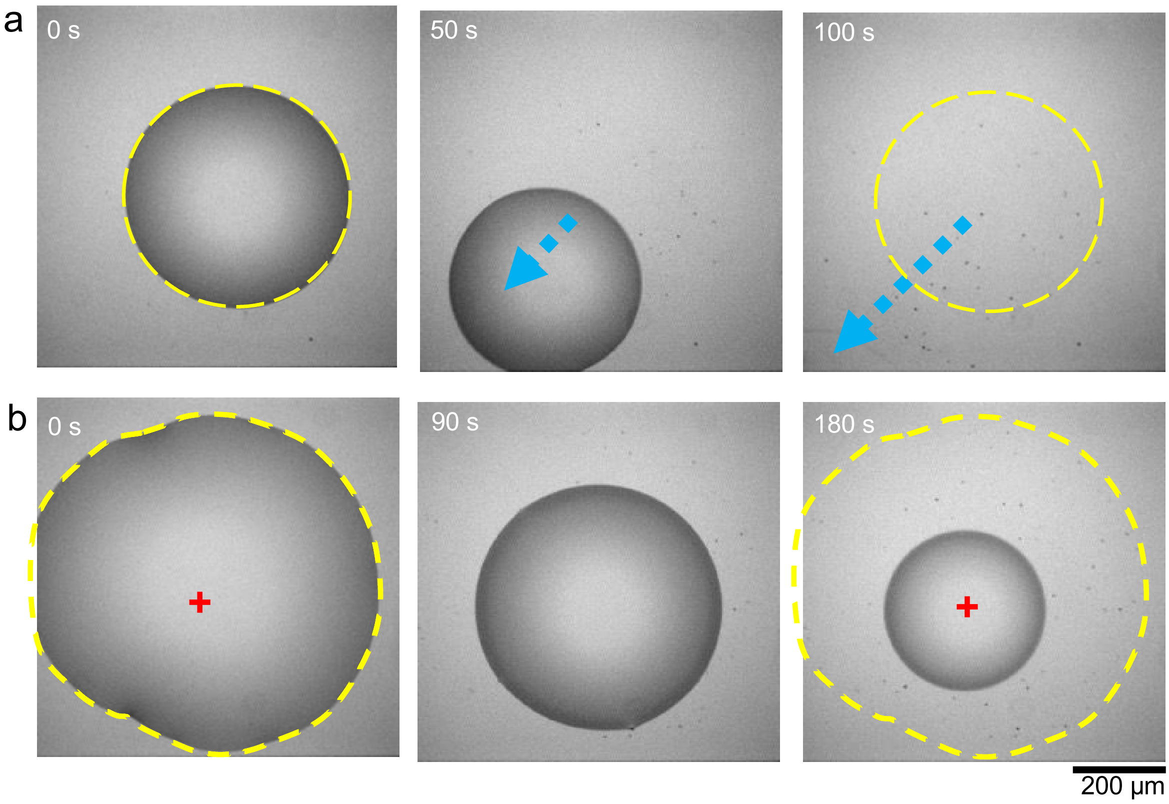

Figure S1: Comparison of sessile droplets undergoing evaporation on a polymer-brushed glass substrate, comparison of a pinned droplet with a self-propelled droplet. a) example of the droplet being self-propelled which moves in the blue arrow trajectory. b) an example of a droplet which contracts towards the center of the mass of the droplet, without showing any self-propulsion.

Contact angle and surface tension measurements

Table S1. Contact angle and surface tension measurements of water and PEG droplets.

Droplet composition	Contact angle on PLL-g-PEG coating	Surface Tension (mN/m)
Water	22	69.03±0.5
PEG 3%	11	59.95±0.15
PEG 1%	13	60.3±0.22

Contraction ratio of the droplet in relation to PEG chain length

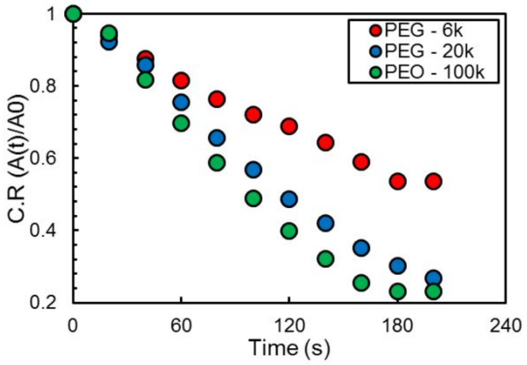

Figure S2: Contraction ratio of the droplets for different PEG molecular weights such as PEO 100k, PEG 20 and 6k. The contraction ratio is measured in the case of the non-propelling droplets by measuring the surface area of the droplet over time A(t) with respect to the initial surface area A_0. The contraction is overall non-linear and the ratio increases with increasing the molecular weight of the surfactant.

Internal flow in a 2D flattened pinned droplet

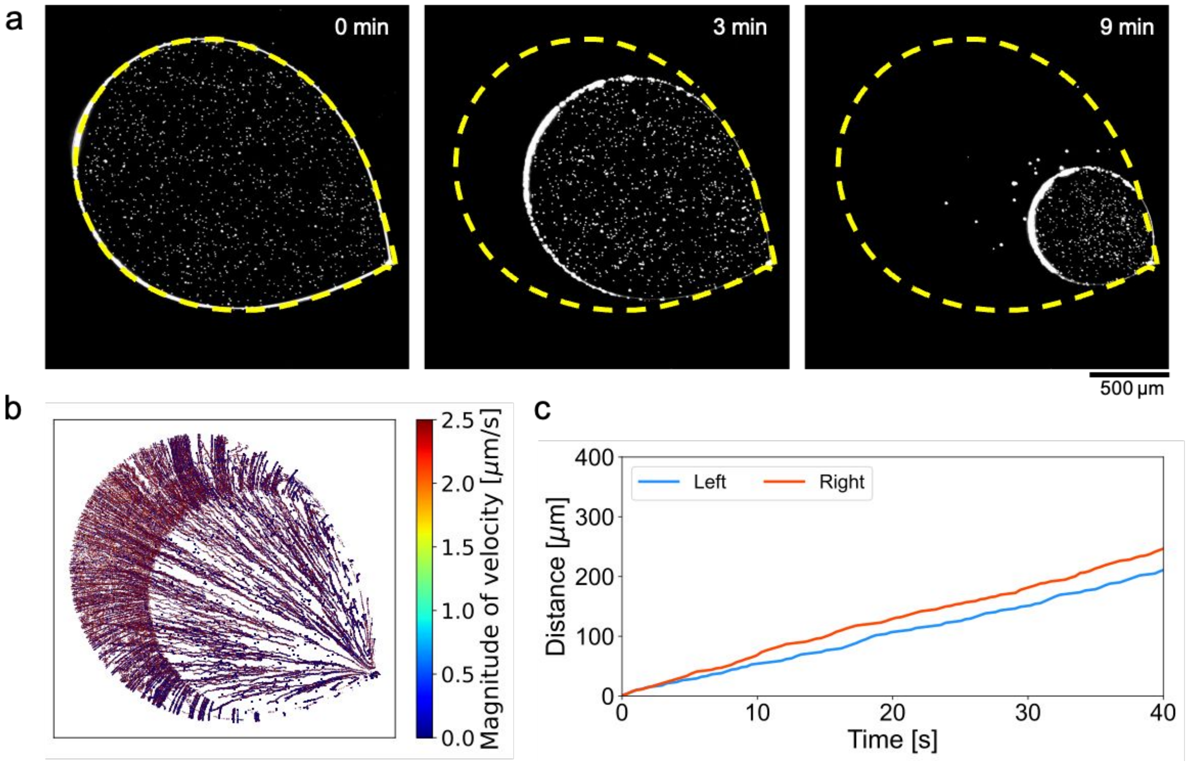

Figure S3: Internal flow in a flattened, pinned 2D droplet that contracts between two polymer-brushed glass substrates during the drying process. a) Time-lapse images of the contracting pinned droplet. b) Particle trajectories colored by velocity magnitude in the contracting pinned droplet, showing the recirculation of particles at the pinning point and the formation of two vortices. c) Measurements of the displacement of the particles at the droplet interface from the pinning point to the back of the droplet.

List of the supporting videos

Video S1: Asymmetry in the strength of the Marangoni vortex in a self-propelled droplet. The vortices that can be seen are a forward and backward circulation of the particles in the band region, which is strong on the back of the self-propelled droplet.
Video S2: Streamlines of the flow inside a self-propelled, sessile droplet.

Video S3: Time-lapse images of the 2D-flattened droplet self-propelling between two surfaces. The particles at the interface flow backwards to the back side of the droplet.
Video S4: Streamlines of the flow inside a self-propelled, 2D flattened droplet.

Video S5: Time-lapse images of a pinned 2D-flattened droplet contracting between two surfaces. Particles at the interface flow backwards to the contracting end of the droplet.
